# Investigation of Oral Health Awareness and Associated Factors Among Japanese University Students: Analyzing Behaviors Influencing Lifelong Oral Health Promotion

**DOI:** 10.3390/healthcare13121370

**Published:** 2025-06-07

**Authors:** Tsukasa Yamamoto, Manato Seguchi, Yukihiro Mori, Harumi Ejiri, Mamoru Tanaka, Hana Kozai, Yoko Iio, Yuka Aoyama, Morihiro Ito

**Affiliations:** 1 Graduate School of Life and Health Sciences, Chubu University, 1200 Matsumoto-cho, Kasugai-shi 487-8501, Aichi, Japan; yama-moto3@fsc.chubu.ac.jp (T.Y.); rb24801-4974@sti.chubu.ac.jp (M.S.); aoyamay@fsc.chubu.ac.jp (Y.A.); 2 Center for Nursing Practicum Support, Chubu University, 1200 Matsumoto-cho, Kasugai 487-8501, Aichi, Japan; 3 Support for Pioneering Research Initiated by the Next Generation (SPRING), Chubu University, 1200 Matsumoto-cho, Kasugai-shi 487-8501, Aichi, Japan; 4 Department of Nursing, College of Life and Health Sciences, Chubu University, 1200 Matsumoto-cho, Kasugai-shi 487-8501, Aichi, Japan; moriyuki0821@fsc.chubu.ac.jp (Y.M.); h-ejiri@fsc.chubu.ac.jp (H.E.); 5 Department of Food and Nutritional Sciences, College of Bioscience and Biotechnology, Chubu University, 1200 Matsumoto-cho, Kasugai-shi 487-8501, Aichi, Japan; m-tanaka@fsc.chubu.ac.jp (M.T.); hkozai@fsc.chubu.ac.jp (H.K.); 6 Department of Lifelong Sports and Health Sciences, College of Life and Health Sciences, Chubu University, 1200 Matsumoto-cho, Kasugai-shi 487-8501, Aichi, Japan; yoks@fsc.chubu.ac.jp; 7 Department of Clinical Engineering, College of Life and Health Sciences, Chubu University, 1200 Matsumoto-cho, Kasugai-shi 487-8501, Aichi, Japan; 8 Department of Biomedical Sciences, College of Life and Health Sciences, Chubu University, 1200 Matsumoto-cho, Kasugai-shi 487-8501, Aichi, Japan

**Keywords:** tooth brushing, dental caries, periodontal disease, systemic diseases

## Abstract

**Background**: University students’ awareness of oral health plays an important role in lifelong health promotion. However, the factors influencing this awareness among Japanese university students are not fully understood. This study aimed to comprehensively examine and analyze Japanese university students’ perceptions of their oral health status, self-reported oral symptoms, and oral health-related behaviors. **Methods**: A cross-sectional survey was conducted among undergraduate students using an anonymous online questionnaire to collect information on their basic attributes and self-reported items related to oral health status, oral health behavior, and lifestyle habits. The chi-square test and logistic regression analysis were used to examine factors associated with oral health status. **Results**: A total of 5482 students participated in this study. Overall, 75.9% of the respondents reported that their oral health was good. Factors significantly associated with good oral health were the absence of dental caries and periodontal disease, tooth brushing at least twice a day, regular dental visits, conscious toothpaste selection, and lack of concern about dental care costs and pain during treatment. **Conclusions**: Oral diseases and symptoms, oral health behaviors, and psychosocial factors were strongly associated with university students’ awareness of their oral health. Since oral health is closely related to systemic health, it is essential to promote proper oral hygiene practices at an early age. Therefore, providing oral health education for university students may contribute to lifelong health promotion and prevention of systemic diseases.

## 1. Introduction

Overall health and well-being are greatly influenced by dental health, which is linked to oral health awareness and good oral health habits [1,2]. Maintaining good oral health has been shown to improve a person’s appearance and mood [3]. The World Health Organization reported that approximately 2 billion people (about 29% of the world’s population) have permanent dental caries and that despite significant advances in population-level oral health measures, oral health issues remain inadequately managed globally [4]. This finding can be attributed to the rapid onset of oral diseases following lifestyle changes, such as a high-sugar diet, consumption of unfluoridated water, and other socio-environmental factors [4]. Oral health is an important public health concern because of the high incidence and prevalence of oral diseases worldwide. Furthermore, in most developed countries, the treatment of oral diseases ranks as the fourth most expensive healthcare expenditure [5].

Dental caries and periodontal disease are the leading oral diseases worldwide and are the two leading causes of tooth loss [6]. The global prevalence of periodontal disease is estimated to be 19%, with over 1 billion cases [4]. Since the 1980s, research on the association between periodontal and systemic diseases has been ongoing [7]. Periodontal disease is a risk factor for systemic diseases, such as diabetes [8,9] and cardiovascular disease [10], and preventive measures, including lifestyle changes, are required. Among oral health issues, rapid teeth deterioration and periodontal disease in young adults are matters of concern. The prevalence of dental caries peaks at 25 years of age [11], and the prevalence of periodontal disease increases in the early 20s [12]. Surveys among Japanese university students have reported that those with more oral health knowledge have better oral health behaviors [13,14].

In Japan, annual oral health examinations are mandatory for students in elementary classes up to high school under the School Health and Safety Law. Subsequently, periodic oral examinations and oral health examinations are the individual’s responsibility. Therefore, students need to be aware of their oral health status during their university years to prevent lifestyle-related diseases. Oral healthcare is essential for maintaining both oral and overall health, and disease prevention is more efficient and cost-effective than treatment after onset. Therefore, oral health awareness is paramount for preventing systemic diseases.

In particular, in Japan, since mandatory oral health checkups are no longer required for university students, these young adults are likely to be left unaware of their oral health status. Therefore, determining the current state of oral health awareness during the transitional period of university education and its association with oral health behavior is an urgent public health issue.

Although reports from other countries [15] have addressed the influence of oral health knowledge, attitudes, and habits on oral health awareness among university students, few comprehensive surveys have evaluated the awareness of oral health status and its factors among Japanese university students. This study aimed to comprehensively investigate and analyze the awareness of oral health status, self-reported oral symptoms, and oral health behavior among Japanese university students. By clarifying the relationship between oral health awareness, lifestyle, and behavior during this transitional stage, when exposure to oral healthcare is limited, we aim to support the lifelong prevention of dental diseases, consequently reducing the risk of systemic disease and ultimately promoting overall health.

## 2. Materials and Methods

### 2.1. Study Design and Participants

This cross-sectional study was conducted among undergraduate students at a private university in Aichi, Japan, between March and April 2024. The university has several faculties, including the Schools of Management and Information Sciences, Science and Engineering, Humanities, and Life and Health Sciences. This study included 10,574 undergraduate students enrolled at the university. Among them, 9392 students underwent a health examination at the beginning of the academic year, during which participants were recruited. The survey was conducted anonymously, and consent was deemed to have been given on completion of the distributed questionnaire. Of 5655 students (60.2%) that agreed to participate in the study, 5651 (99.9%) provided valid responses. However, there were later exclusions because the undergraduate students included 169 graduate students. As a result, the final analytic sample consisted of 5482 undergraduate students.

Statistical power analysis was conducted using SPSS version 29 (IBM Corp., Armonk, NY, USA) to determine the sample size required for this study. The results indicated that a sample size of 395 was needed to achieve a power of 0.8, a significance level of 0.05, and 32 explanatory variables. The sample size of this study met this requirement.

This study was approved by the Chubu University Ethics Review Committee (#20230056 and #20220001).

### 2.2. Survey

Data were collected using Google Forms (https://www.google.com/forms/about/), Last accessed on 1 May 2025. Participants completed the survey anonymously.

#### 2.2.1. Basic Items

Participants provided basic information, such as age, sex, year of study, whether they lived together with family, whether they participated in varsity sports, smoking history, satisfaction with current life [16], and how they felt about their overall health [17].

#### 2.2.2. Oral Health

Participants were asked to select either good or poor to describe how they perceived their oral health.

Similar to the studies by Ishikawa et al. [18] and Rana et al. [19], who examined awareness of oral diseases and symptoms as survey items, we asked questions that facilitated self-reporting of the presence of dental caries and periodontal disease, as indicators of oral diseases, and of bad breath and gum bleeding, as indicators of oral symptoms. The data on oral health were derived entirely from self-reported data and were not clinically validated.

#### 2.2.3. Oral Health Awareness

The oral health awareness questionnaire was designed also to address concerns, knowledge, perceptions, and anxiety about oral health. The questionnaire was adapted from studies by Taniguchi et al., Tadin et al., and Peltzer et al. [14,20,21] who examined the influence of oral knowledge and perceptions of oral health. The questionnaire included questions about interest in oral health and hygiene to determine the level of interest. Regarding knowledge of oral health, we asked questions about the student’s knowledge of the 8020 campaign (this campaign was launched in 1989 by the then Ministry of Health and Welfare and the Japan Dental Association to encourage people to keep at least 20 of their original teeth by the age of 80 years), of the fact that periodontal disease is a lifestyle-related disease, and of the fact that smoking causes periodontal disease. In addition, regarding awareness of oral health, we asked whether the students consciously selected their toothbrushing tools, and whether they brushed their teeth with attention to dental caries and periodontal disease. Furthermore, the questionnaire included questions on concerns about the cost of dental visits or pain caused by dental treatment [22].

#### 2.2.4. Oral Health Behavior

Questions about oral health behavior were adapted from studies that reported on oral health habits [17,23], such as frequency of tooth brushing (once daily or less, twice daily, and three times daily or more) [17], use of mouthwash, use of interdental brushes and dental floss, and regular dental visits. Our survey included questions on the frequency of tooth brushing, use of mouthwash, use of interdental brushes and dental floss, history of dental visits (within 6 months), status of regular dental visits, availability of a family dentist, and receipt of education on oral hygiene.

Since oral health is reportedly influenced by eating habits [24] and type of fluid intake [25], we also asked how often breakfast was consumed (4 days/week or less and 5 days/week or more), how much water was consumed (mL/day), the type of beverage frequently consumed, and whether they usually chewed gum.

### 2.3. Statistical Analysis

SPSS, version 29, (IBM Corp., Armonk, NY, USA) was used for statistical analysis. Pearson’s chi-square test was used to analyze the association between multiple explanatory variables, such as awareness of oral diseases/symptoms, oral health, oral health behavior, and oral health status. Similarly, Pearson’s chi-square test was used to analyze the association between the above explanatory variables and the student’s year of study.

Next, binomial logistic regression analysis using forward selection was performed with only those variables that showed statistically significant associations in the chi-square test. Adjusted odds ratios (AORs) and 95% confidence intervals (95% CIs) were calculated for each explanatory variable. The calculated factors were sorted in order of increasing AORs. Statistical significance was set at *p* < 0.05.

## 3. Results

### 3.1. Study Participants

Participants’ characteristics are shown in Table 1. After exclusion, the analysis included 5482 participants (mean age, 19.32 years; standard deviation 1.26 years). The participants were 3693 (67.4%) men and 1752 (32.0%) women, and 37 (0.7%) did not answer, with the majority being first-year students (2176, 39.7%). In addition, 4623 (84.3%) participants lived with family or relatives, 720 (13.1%) participated in varsity sports, and 382 (7.0%) had a smoking history. Furthermore, 4031 (73.5%) participants indicated that they were satisfied with their current lives, and 4975 (90.8%) reported that their overall health was good.

### 3.2. Factors Associated with Basic Attributes and Oral Health Status

Regarding their oral health status, 4159 (75.9%) students reported good status and 1323 (24.1%) reported poor status. The differences in the basic attributes and oral health status between the groups are shown in Table 2. The good oral health group had a significantly higher percentage of respondents who were satisfied with their current lives and reported good overall health (*p* < 0.001). However, no significant differences between age, sex, year of study, living with family, participation in varsity sports, and smoking history were observed between the groups. Additionally, no significant differences were observed among all faculties.

### 3.3. Factors Associated with Oral Diseases and Symptoms and Oral Health Status

The differences in oral diseases, symptoms, and oral health status between the groups are shown in Table 3. The good oral health group had a significantly higher percentage of respondents who reported no dental caries, periodontal disease, bad breath, or gum bleeding (*p* < 0.001).

### 3.4. Factors Associated with Oral Health Awareness and Oral Health Status

The differences in oral health awareness between the groups are shown in Table 4. The good oral health group had a significantly higher percentage of students who knew about the 8020 campaign (*p* = 0.003), that smoking causes periodontal disease, consciously selected toothbrushing tools, were mindful of dental caries and periodontal disease during toothbrushing, and were not concerned about the cost of dental visits and pain during dental treatment than the poor oral health group (all *p* < 0.001). However, no significant differences were observed in interest in oral health, interest in oral hygiene, or knowledge that periodontal disease is related to lifestyle.

### 3.5. Factors Associated with Oral Health Behavior and Oral Health Status

The differences in oral health behavior between the groups are shown in Table 5. The good oral health group had a significantly higher proportion of students who used mouthwash (*p* < 0.001) and had regular dental visits (*p* = 0.004). The poor oral health group was significantly more likely to report that they brushed their teeth once or less daily (*p* < 0.001) and received oral hygiene education (*p* = 0.003). No significant differences were observed in the use of interdental brushes and dental floss, history of a dental visit (within 6 months), and availability of a family dentist.

Regarding eating habits, only the good oral health group had a significantly higher percentage of respondents reporting that they consumed breakfast more frequently than 5 days/week (*p* < 0.001). However, no significant differences were noted in fluid intake (mL/day), type of beverage frequently consumed, and frequent chewing of gum.

### 3.6. Factors Associated with Oral Health Behavior and Year of Study

The differences in oral health behavior according to the year of study are presented in Table 6. Significant differences were found between year of study and sex (*p* < 0.001), smoking history (*p* < 0.001), satisfaction with current life (*p* < 0.001), and overall health status (*p* < 0.001). Interest in oral health (*p* < 0.001), conscious selection of toothbrushing tools (*p* < 0.001), and anxiety about the pain associated with dental treatment (*p* = 0.010) were also significantly different according to the year of study. In addition, toothbrushing frequency (*p* < 0.001), dental visits within the previous 6 months (*p* < 0.001), regular dental visits (*p* < 0.001), having a family dentist (*p* = 0.003), frequency of breakfast intake (*p* < 0.001), and frequent gum chewing (*p* < 0.001) were significantly different among the students, depending on the year of study.

### 3.7. Logistic Regression Analysis

Table 7 shows the results of the logistic regression analyses. The positive predictors of oral health status included no dental caries (OR = 6.253, 95% CI: 4.952–7.897, *p* < 0.001), no periodontal disease (OR = 4.476, 95% CI: 3.083–6.497, *p* < 0.001), good overall health (OR = 2.493, 95% CI: 2.011–3.092, *p* < 0.001), no gum bleeding (OR = 1.834, 95% CI: 1.588–2.117, *p* < 0.001), and no bad breath (OR = 1.829, 95% CI: 1.584–2.111, *p* < 0.001). Brushing teeth three times daily or more (reference: once daily or less) (OR = 1.745, 95% CI: 1.386–2.195, *p* < 0.001) or twice daily (reference: once daily or less) (OR = 1.604, 95% CI: 1.336–1.924, *p* < 0.001), consciously selecting brushing tools (OR = 1.466, 95% CI: 1.230–1.746, *p* < 0.001), being aware of caries and periodontal disease (OR = 1.378, 95% CI: 1.181–1.608, *p* < 0.001), having breakfast 5 days/week or more (OR = 1.261, 95% CI: 1.091–1.458, *p* = 0.002), use of mouthwash (OR = 1.255, 95% CI: 1.022–1.540, *p* = 0.030), satisfaction with current life (OR = 1.251, 95% CI: 1.073–1.459, *p* = 0.004), and regular dental visits (OR = 1.235, 95% CI: 1.049–1.455, *p* = 0.012) were also found to be positive predictors. However, concern about pain during dental treatment (OR = 0.572, 95% CI: 0.482–0.679, *p* < 0.001), concern about the cost of dental visits (OR = 0.769, 95% CI: 0.664–0.890, *p* < 0.001), and having received education on oral hygiene (OR = 0.812, 95% CI: 0.698–0.945, *p* = 0.007) were found to be negative predictors.

## 4. Discussion

A total of 1323 participants (24.1%) reported poor oral health, indicating that approximately one-quarter of the university students had a negative perception of their oral health status. Low oral health literacy may lead to lower self-assessment of oral health status and an increased risk of oral disease [26]. Therefore, educational programs aimed at improving oral health literacy among university students need to be developed and implemented urgently. Previous studies have indicated that oral health behavior is key to building awareness of oral health status [17,23,27]. Therefore, by investigating factors associated with awareness of oral health status among university students, we can identify aspects that require attention and help this population achieve good oral health awareness and maintain overall health.

There is a Japanese study by Nakahara et al. [28] in which the age group and population of the subjects are similar to those of this study, but the proportion of people living alone and living with others in terms of living arrangement is different. Furthermore, students who live with family or relatives reported significantly higher rates of dental visits [28] and greater awareness of oral health conditions than those who did not. In addition, an overseas study by Thirunavukkarasu et al. reported that cultural background differences, such as lifestyle habits and awareness of oral hygiene, may affect the results due to differences in the target countries [29]. Furthermore, smokers were shown to be less aware of their oral health status than nonsmokers [29]. In comparison, in this study, attributes such as sex, household status, and smoking history were not directly related to awareness of oral health status. In addition, sex distribution, household status, and smoking history differed significantly between this study and previous studies [28,29]. This difference may be due to differences in local culture and lifestyle, which may contribute to differences in gender, household status, and percentage of smoking history.

The rates of regular dental examinations and examinations within 6 months decreased as the academic year progressed (Table 6C). This may be because dental checkups are not mandatory for university students; moreover, the financial burden of the checkup may have had an impact [19,30,31]. The rate of dental checkups in this study was lower than the rates reported in the studies by Sumita et al. and Mueller et al., who examined individuals in their early 20s [17,32]. The long-term lack of visits to dentists, owing to low rates of dental checkups and decreased oral health awareness, increases the risk of dental caries and periodontal disease. First-year students had a significantly higher rate of dental visits than those in other years, indicating the need for oral health literacy interventions for students in advanced academic years. Although a few studies have compared dental visits among Japanese university students by year of study, to our knowledge, this study is the first to demonstrate the need for improved oral health awareness to maintain good oral health in the future.

The absence of dental caries, periodontal disease, bad breath, and gum bleeding had a significant impact on the awareness of oral health status (Table 7). Those with high awareness of their oral health status tend to be aware of whether they have oral diseases or symptoms [27,33], consistent with the results of our study; they also exhibit good oral health behaviors that prevent oral diseases or symptoms [34]. Therefore, developing appropriate oral health behaviors that prevent the onset of diseases and symptoms is essential for improving awareness of oral health status.

Good overall health and life satisfaction had a significant impact on oral health awareness (Table 7). Most students with no history of systemic diseases typically have good oral health; therefore, the link between these aspects must be emphasized when providing health education to university students. Furthermore, in a previous study [35], the participants with a high sense of satisfaction with life had few oral symptoms, such as dental caries and bad breath, and enhanced oral health awareness.

Regarding oral health behavior, brushing twice a day or more (especially three times a day or more), using mouthwash, and having regular dental visits had significant positive effects on the awareness of oral health status (Table 7). Oral health behaviors help prevent oral diseases and symptoms [36,37,38]. Therefore, educational interventions promoting proper oral health behaviors are essential.

Conscious selection of toothbrushing tools and paying attention to caries and periodontal disease had a significant impact on the awareness of oral health status (Table 7). The importance of conscious selection of dental cleaning and toothbrushing tools to maintain oral health status has been reported previously [39,40]. Knowledge of appropriate tools and toothbrushing methods is important for removing oral plaque and preventing dental caries and periodontal disease; hence, these aspects should be included in educational content aimed at promoting good oral health awareness.

Having breakfast five days a week or more had a significant impact on the awareness of oral health status (Table 7). Healthy lifestyle habits [41] such as eating breakfast and knowledge of eating habits impact the risk of developing oral diseases [24]; skipping breakfast may cause periodontal disease [42]. Developing healthy eating habits should be considered necessary educational content for good oral health awareness.

Anxiety about pain during dental treatment and the cost of dental visits had a significant negative impact on the awareness of oral health status (Table 7). These anxieties prevent people from visiting the dentist [19,30,31], resulting in decreased oral health awareness. Thus, it is necessary to provide information regarding the benefits of the proper diagnosis and treatment of oral diseases.

To our knowledge, no study has reported an association between the history of oral hygiene instruction and students’ own perceptions of their oral health status. In the present study, students who had received oral hygiene instruction tended to have significantly more negative perceptions of their oral health status. As suggested in the study by Nakahara et al. [28], oral hygiene education is often provided during dental visits. The findings of oral problems such as tartar and dental caries could lead to negative perceptions of one’s own oral condition as unhealthy. Furthermore, at the time of university enrollment, more than 30% of students already exhibit symptoms of periodontal disease [43]. Additionally, among individuals aged 40 years and older, the prevalence of periodontal disease—which has been linked to systemic diseases—exceeds 40% in men and 30% in women [44]. Increased awareness of oral symptoms [45,46] and oral health education have been reported to be effective in preventing oral diseases and symptoms [47]. Therefore, oral health education is urgently needed to help young adult students maintain oral health. Based on the findings of this study, we believe that education on oral diseases and symptoms and their impact on health should be provided immediately after students enter college, when dental examinations are no longer mandatory. In addition, education on the importance of oral health behaviors, such as frequency of tooth brushing, selection of oral hygiene products, and appropriate brushing methods, should be provided to promote general oral health and prevent the onset of oral diseases. Furthermore, it would be an effective strategy for promoting long-term oral health.

A limitation of this study is that it was conducted at a single university, and selection bias may have been introduced by the absence of students who declined to participate in the study. Second, the oral health status in this study was based on subjective perceptions and awareness and not on objective diagnostic measurements of oral health status. Furthermore, although the university in question was coeducational, the participants were predominantly male. Therefore, while the results of this study may not be generalizable, our data must be considered in future studies on the oral hygiene behaviors of college students.

Finally, oral health problems have been attributed to the elimination of mandatory dental examinations for university students. Compared to the rate of dental examinations in the study by Zimmermann et al. [36], the low rate in this study is remarkable, and to our knowledge, this is the first study to demonstrate the relationship between the rate of dental examinations and the academic year. The government of our country is continuing its 8020 campaign to promote the goal of retaining at least 20 of one’s own teeth by the age of 80, emphasizing that having 20 or more teeth is closely related to proper chewing, adequate nutritional intake, and improved quality of life for the elderly. The 8020 campaign has played a central role in shaping Japan’s oral health policy, in the development of preventive dentistry, and in the formulation of a comprehensive public oral health strategy. The findings of our study could be used to design new health prevention measures for promoting students’ oral health behavior.

## 5. Conclusions

The absence of dental caries and periodontal disease and oral health behaviors, such as frequent toothbrushing, were the factors that contributed to favorable perceptions of oral health status among Japanese university students. The number of dental visits decreased with the progressing academic year. The low level of awareness and recognition of oral health among university students was related to an increased risk of oral and, eventually, systemic diseases; therefore, university students must be educated about oral health. Although this study was conducted at a single university, it is significant in that it comprehensively clarified self-perceived oral health status and its associated factors through a large-scale survey of Japanese university students. Another important feature of this study is its focus on the transition period of university life, during which mandatory dental checkups are no longer provided. These findings highlight the need for education and intervention programs to help university students maintain oral and systemic health. They also provide essential evidence for the development of effective health promotion strategies in terms of preventive medicine and public health. Effective health promotion measures based on empirical evidence from longitudinal and interventional studies need to be developed and disseminated.

## Figures and Tables

**Table 1:** Summary of participant characteristics.

				***n*** ** = 5482**
		***n***		**%**
Mean age ± SD	19.32 ± 1.26			
Sex				
Male		3693		67.4%
Female		1752		32.0%
No answer		37		0.7%
Year of study				
First year		2176		39.7%
Second year		1175		21.4%
Third year		1121		20.5%
Fourth year		1010		18.4%
Living together with family				
Living together		4623		84.3%
Not living together		859		15.7%
Varsity sport participation				
Yes		720		13.1%
No		4762		86.9%
Smoking history				
Yes		382		7.0%
No		5100		93.0%
Satisfaction with current life				
Satisfied		4031		73.5%
Not satisfied		1451		26.5%
Overall health				
Good		4975		90.8%
Poor		507		9.2%

SD, standard deviation.

**Table 2:** Relationship between attributes and oral health.

Variables	All		Oral Health				*p*-Value
			Poor *n* = 1323, 24.1%		Good *n* = 4159, 75.9%		
	*n*	%	*n*	%	*n*	%	
**Attribute**							
Age							
18–19 years	3215	58.6%	769	58.1%	2446	58.8%	n.s.
21 years and older	2267	41.4%	554	41.9%	1713	41.2%	
Sex							
Male	3693	67.4%	901	68.1%	2792	67.1%	n.s.
Female	1752	32.0%	414	31.3%	1338	32.2%	
No answer	37	0.7%	8	0.6%	29	0.7%	
Year of study							
First year	2176	39.7%	495	37.4%	1681	40.4%	n.s.
Second year	1175	21.4%	306	23.1%	869	20.9%	
Third year	1121	20.5%	284	21.5%	837	20.1%	
Fourth year	1010	18.4%	238	18.0%	772	18.6%	
Living together with family							
Living together	4623	84.3%	1127	85.2%	3496	84.1%	n.s.
Not living together	859	15.7%	196	14.8%	663	15.9%	
Varsity sport participation							
Yes	720	13.1%	183	13.8%	537	12.9%	n.s.
No	4762	86.9%	1140	86.2%	3622	87.1%	
Smoking history							
Yes	382	7.0%	98	7.4%	284	6.8%	n.s.
No	5100	93.0%	1225	92.6%	3875	93.2%	
Satisfaction with current life							
Satisfied	4031	73.5%	868	65.6%	3163	76.1%	<0.001
Not satisfied	1451	26.5%	455	34.4%	996	23.9%	
Overall health							
Good	4975	90.8%	1083	81.9%	3892	93.6%	<0.001
Poor	507	9.2%	240	18.1%	267	6.4%	

The *p*-values were calculated using a chi-square test. n.s., no significant difference.

**Table 3:** Relationship between oral diseases and symptoms and oral health status.

Variables	All		Oral Health				*p*-Value
			Poor *n* = 1323, 24.1%		Good *n* = 4159, 75.9%		
	*n*	%	*n*	%	*n*	%	
**Oral Diseases and Symptoms**							
Dental caries							
Yes	421	7.7%	269	20.3%	152	3.7%	<0.001
No	5061	92.3%	1054	79.7%	4007	96.3%	
Periodontal disease							
Yes	152	2.8%	98	7.4%	54	1.3%	<0.001
No	5330	97.2%	1225	92.6%	4105	98.7%	
Bad breath							
Yes	2121	38.7%	747	56.5%	1374	33.0%	<0.001
No	3361	61.3%	576	43.5%	2785	67.0%	
Gum bleeding							
Yes	2045	37.3%	717	54.2%	1328	31.9%	<0.001
No	3437	62.7%	606	45.8%	2831	68.1%	

The *p*-values were calculated using a chi-square test.

**Table 4:** Relationship between awareness of oral health and oral health status.

Variables	All		Oral Health				*p*-Value
			Poor *n* = 1323, 24.1%		Good *n* = 4159, 75.9%		
	*n*	%	*n*	%	*n*	%	
**Awareness of Oral Health**							
Interest in oral health							
Yes	3960	72.2%	970	73.3%	2990	71.9%	n.s.
No	1522	27.8%	353	26.7%	1169	28.1%	
Interest in oral hygiene							
Yes	4468	81.5%	1059	80.0%	3409	82.0%	n.s.
No	1014	18.5%	264	20.0%	750	18.0%	
Knowledge of the 8020 campaign							
Yes	3246	59.2%	737	55.7%	2509	60.3%	0.003
No	2236	40.8%	586	44.3%	1650	39.7%	
Knowledge that periodontal disease is a lifestyle disease							
Yes	4272	77.9%	1017	76.9%	3255	78.3%	n.s.
No	1210	22.1%	306	23.1%	904	21.7%	
Knowledge of the impact of smoking on periodontal disease development							
Yes	4302	78.5%	981	74.1%	3321	79.9%	<0.001
No	1180	21.5%	342	25.9%	838	20.1%	
Consciously selected toothbrushing tools							
Yes	1717	31.3%	303	22.9%	1414	34.0%	<0.001
No	3765	68.7%	1020	77.1%	2745	66.0%	
Tooth brushing with attention to caries and periodontal disease							
Yes	3454	63.0%	731	55.3%	2723	65.5%	<0.001
No	2028	37.0%	592	44.7%	1436	34.5%	
Anxiety about the cost of dental visits							
Yes	1995	36.4%	637	48.1%	1358	32.7%	<0.001
No	3487	63.6%	686	51.9%	2801	67.3%	
Anxiety about pain during dental treatment							
Yes	995	18.2%	386	29.2%	609	14.6%	<0.001
No	4487	81.8%	937	70.8%	3550	85.4%	

The *p*-values were calculated using a chi-square test. n.s., no significant difference.

**Table 5:** Relationship between oral health behavior and oral health status.

Variables	All		Oral Health				*p*-Value
			Poor *n* = 1323, 24.1%		Good *n* = 4159, 75.9%		
	*n*	%	*n*	%	*n*	%	
**Oral Health Habits**							
Frequency of tooth brushing (times/day)							
0–1 times/day	828	15.1%	327	24.7%	501	12.0%	<0.001
2 times/day	3463	63.2%	787	59.5%	2676	64.3%	
3 times/day or more	1191	21.7%	209	15.8%	982	23.6%	
Use of mouthwash							
Yes	904	16.5%	174	13.2%	730	17.6%	<0.001
No	4578	83.5%	1149	86.8%	3429	82.4%	
Use of interdental brush and dental floss							
Yes	856	15.6%	184	13.9%	672	16.2%	n.s.
No	4626	84.4%	1139	86.1%	3487	83.8%	
Dental visit (within 6 months)							
Yes	2170	39.6%	514	38.9%	1656	39.8%	n.s.
No	3312	60.4%	809	61.1%	2503	60.2%	
Regular dental visits							
Yes	1540	28.1%	331	25.0%	1209	29.1%	0.004
No	3942	71.9%	992	75.0%	2950	70.9%	
Availability of a family dentist							
Yes	3039	55.4%	717	54.2%	2322	55.8%	n.s.
No	2443	44.6%	606	45.8%	1837	44.2%	
Whether the patient has received education on oral hygiene							
Yes	3485	63.6%	886	67.0%	2599	62.5%	0.003
No	1997	36.4%	437	33.0%	1560	37.5%	
**Eating Habits**							
Frequency of breakfast intake (days/week)							
More than 5 days	3603	65.7%	774	58.5%	2829	68.0%	<0.001
Less than 4 days	1879	34.3%	549	41.5%	1330	32.0%	
Water intake (mL/day)							
Less than 1000 mL	2986	54.5%	730	55.2%	2256	54.2%	n.s.
1000 mL or more	2496	45.5%	593	44.8%	1903	45.8%	
Types of beverages most often consumed							
Water	2747	50.1%	645	48.8%	2102	50.5%	n.s.
Green tea	2506	45.7%	596	45.0%	1910	45.9%	
Soft drinks	229	4.2%	82	6.2%	147	3.5%	
Frequent gum chewing							
Yes	799	14.6%	183	13.8%	616	14.8%	n.s.
No	4683	85.4%	1140	86.2%	3543	85.2%	

The *p*-values were calculated using a chi-square test. n.s., no significant difference.

**Table 6:** (**A**) Oral health behavior—comparison by year of study. (**B**) Oral health diseases or oral health outcomes—comparison by year of study. (**C**) Oral health behavior—comparison by year of study.

Variables	All		Year of Study								*p*-Value
			First Year *n* = 2176, 39.7%		Second Year *n* = 1175, 21.4%		Third Year *n* = 1121, 20.5%		Fourth Year *n* = 1010, 18.4%		
	*n*	%	*n*	%	*n*	%	*n*	%	*n*	%	
(**A**)											
**Attribute**											
Sex											
Male	3693	67.4%	1497	68.8%	832	70.8%	734	65.5%	630	62.4%	<0.001
Female	1752	32.0%	668	30.7%	335	28.5%	379	33.8%	370	36.6%	
No answer	37	0.7%	11	0.5%	8	0.7%	8	0.7%	10	1.0%	
Living together with family											
Not Living together	859	15.7%	309	14.2%	193	16.4%	196	17.5%	161	15.9%	n.s.
Living together	4623	84.3%	1867	85.8%	982	83.6%	925	82.5%	849	84.1%	
Varsity sport participation											
Yes	720	13.1%	288	13.2%	133	11.3%	163	14.5%	136	13.5%	n.s.
No	4762	86.9%	1888	86.8%	1042	88.7%	958	85.5%	874	86.5%	
Smoking history											
Yes	382	7.0%	9	0.4%	27	2.3%	155	13.8%	191	18.9%	<0.001
No	5100	93.0%	2167	99.6%	1148	97.7%	966	86.2%	819	81.1%	
Satisfaction with current life											
Satisfied	4031	73.5%	2027	93.2%	992	84.4%	474	42.3%	538	53.3%	<0.001
Not satisfied	1451	26.5%	149	6.8%	183	15.6%	647	57.7%	472	46.7%	
Overall health											
Good	4975	90.8%	2030	93.3%	1053	89.6%	976	87.1%	916	90.7%	<0.001
Poor	507	9.2%	146	6.7%	122	10.4%	145	12.9%	94	9.3%	
(**B**)											
**Oral Health**											
Oral health											
Yes	4159	75.9%	1681	77.3%	869	74.0%	837	74.7%	772	76.4%	n.s.
No	1323	24.1%	495	22.7%	306	26.0%	284	25.3%	238	23.6%	
Dental caries											
Yes	421	7.7%	169	7.8%	93	7.9%	71	6.3%	88	8.7%	n.s.
No	5061	92.3%	2007	92.2%	1082	92.1%	1050	93.7%	922	91.3%	
Periodontal disease											
Yes	152	2.8%	47	2.2%	35	3.0%	35	3.1%	35	3.5%	n.s.
No	5330	97.2%	2129	97.8%	1140	97.0%	1086	96.9%	975	96.5%	
Bad breath											
Yes	2121	38.7%	825	37.9%	488	41.5%	432	38.5%	376	37.2%	n.s.
No	3361	61.3%	1351	62.1%	687	58.5%	689	61.5%	634	62.8%	
Gum bleeding											
Yes	2045	37.3%	810	37.2%	449	38.2%	421	37.6%	365	36.1%	n.s.
No	3437	62.7%	1366	62.8%	726	61.8%	700	62.4%	645	63.9%	
**Awareness of Oral Health**											
Interests in oral health											
Yes	3960	72.2%	1522	69.9%	836	71.1%	819	73.1%	783	77.5%	<0.001
No	1522	27.8%	654	30.1%	339	28.9%	302	26.9%	227	22.5%	
Interest in oral hygiene											
Yes	4468	81.5%	1755	80.7%	951	80.9%	925	82.5%	837	82.9%	n.s.
No	1014	18.5%	421	19.3%	224	19.1%	196	17.5%	173	17.1%	
Knowledge of the 8020 campaign											
Yes	3246	59.2%	1335	61.4%	669	56.9%	657	58.6%	585	57.9%	n.s.
No	2236	40.8%	841	38.6%	506	43.1%	464	41.4%	425	42.1%	
Knowledge that periodontal disease is a lifestyle disease											
Yes	4272	77.9%	1682	77.3%	929	79.1%	883	78.8%	778	77.0%	n.s.
No	1210	22.1%	494	22.7%	246	20.9%	238	21.2%	232	23.0%	
Knowledge of the impact of smoking on periodontal disease development											
Yes	4302	78.5%	1678	77.1%	950	80.9%	875	78.1%	799	79.1%	n.s.
No	1180	21.5%	498	22.9%	225	19.1%	246	21.9%	211	20.9%	
Consciously selected toothbrushing tools											
Yes	1717	31.3%	619	28.4%	373	31.7%	359	32.0%	366	36.2%	<0.001
No	3765	68.7%	1557	71.6%	802	68.3%	762	68.0%	644	63.8%	
Brushing teeth with attention to caries and periodontal disease											
Yes	3454	63.0%	1339	61.5%	753	64.1%	715	63.8%	647	64.1%	n.s.
No	2028	37.0%	837	38.5%	422	35.9%	406	36.2%	363	35.9%	
Anxiety about the cost of dental visits											
Yes	1995	36.4%	773	35.5%	418	35.6%	428	38.2%	376	37.2%	n.s.
No	3487	63.6%	1403	64.5%	757	64.4%	693	61.8%	634	62.8%	
Anxiety about pain during dental treatment											
Yes	995	18.2%	353	16.2%	223	19.0%	233	20.8%	186	18.4%	0.010
No	4487	81.8%	1823	83.8%	952	81.0%	888	79.2%	824	81.6%	
(**C**)											
**Oral Health Habits**											
Frequency of toothbrushing (times/day)											
0–1 times/day	828	15.1%	283	13.0%	182	15.5%	189	16.9%	174	17.2%	<0.001
2 times/day	3463	63.2%	1422	65.3%	758	64.5%	698	62.3%	585	57.9%	
3 times/day or more	1191	21.7%	471	21.6%	235	20.0%	234	20.9%	251	24.9%	
Use of mouthwash											
Yes	904	16.5%	323	14.8%	207	17.6%	194	17.3%	180	17.8%	n.s.
No	4578	83.5%	1853	85.2%	968	82.4%	927	82.7%	830	82.2%	
Use of interdental brush and dental floss											
Yes	856	15.6%	315	14.5%	187	15.9%	176	15.7%	178	17.6%	n.s.
No	4626	84.4%	1861	85.5%	988	84.1%	945	84.3%	832	82.4%	
Dental visits (within 6 months)											
Yes	2170	39.6%	1001	46.0%	420	35.7%	395	35.2%	354	35.0%	<0.001
No	3312	60.4%	1175	54.0%	755	64.3%	726	64.8%	656	65.0%	
Regular dental visits											
Yes	1540	28.1%	677	31.1%	321	27.3%	288	25.7%	254	25.1%	<0.001
No	3942	71.9%	1499	68.9%	854	72.7%	833	74.3%	756	74.9%	
Availability of a family dentist											
Yes	3039	55.4%	1272	58.5%	640	54.5%	592	52.8%	535	53.0%	0.003
No	2443	44.6%	904	41.5%	535	45.5%	529	47.2%	475	47.0%	
Experience with oral hygiene education											
Yes	3485	63.6%	1396	64.2%	742	63.1%	724	64.6%	623	61.7%	n.s.
No	1997	36.4%	780	35.8%	433	36.9%	397	35.4%	387	38.3%	
**Eating Habits**											
Frequency of breakfast intake (days/week)											
More than 5 days	3603	65.7%	1643	75.5%	763	64.9%	666	59.4%	531	52.6%	<0.001
Less than 4 days	1879	34.3%	533	24.5%	412	35.1%	455	40.6%	479	47.4%	
Water intake (mL/day)											
Less than 1000 mL	2986	54.5%	1183	54.4%	644	54.8%	623	55.6%	536	53.1%	n.s.
1000 mL or more	2496	45.5%	993	45.6%	531	45.2%	498	44.4%	474	46.9%	
Types of beverages most often consumed											
Water	2747	50.1%	1047	48.1%	604	51.4%	572	51.0%	524	51.9%	n.s.
Green tea	2506	45.7%	1038	47.7%	514	43.7%	508	45.3%	446	44.2%	
Soft drinks	229	4.2%	91	4.2%	57	4.9%	41	3.7%	40	4.0%	
Frequent gum chewing											
Yes	799	14.6%	362	16.6%	227	19.3%	194	17.3%	16	1.6%	<0.001
No	4683	85.4%	1814	83.4%	948	80.7%	927	82.7%	994	98.4%	

The *p*-values were calculated using a chi-square test. n.s., no significant difference.

**Table 7:** Factors affecting the awareness of oral health status.

	OR	OR 95% CI		*p*-Value
		Lower Limit	Upper Limit	
Predictors of Oral Health Status				
No dental caries	6.253	4.952	7.897	<0.001
No periodontal disease	4.476	3.083	6.497	<0.001
Good overall health	2.493	2.011	3.092	<0.001
No gum bleeding	1.834	1.588	2.117	<0.001
No bad breath	1.829	1.584	2.111	<0.001
Brushing teeth 3 times/day or more (reference: 1 time/day or less) ⁑	1.745	1.386	2.195	<0.001
Brushing teeth twice/day (reference: 1 time/day or less) ⁑	1.604	1.336	1.924	<0.001
Conscious selection of toothbrushing tools	1.466	1.230	1.746	<0.001
Brushing teeth with attention to caries and periodontal disease	1.378	1.181	1.608	<0.001
Consuming breakfast more than 5 days/week	1.261	1.091	1.458	0.002
Use of mouthwash	1.255	1.022	1.540	0.030
Satisfaction with present life	1.251	1.073	1.459	0.004
Regular dental visits	1.235	1.049	1.455	0.012
Experience with oral hygiene education	0.812	0.698	0.945	0.007
Anxiety about the cost of dental visits	0.769	0.664	0.890	<0.001
Anxiety about pain during dental treatment	0.572	0.482	0.679	<0.001

The *p*-values were calculated using binary logistic regression analysis. Perception of oral health status (good = 1, poor = 0) was the dependent variable. Frequency of tooth brushing (0–1 times/day = 0, 2 times/day = 1, 3 or more times/day = 2), frequency of breakfast eating (5 or more days/week = 1, less than 4 days/week = 0), satisfaction with current life (satisfied = 1, not satisfied = 0), and other (yes = 1, no = 0) were the explanatory variables. OR: odds ratio; CI: confidence interval. ⁑, vs. 1 time/day.

## Data Availability

The data presented in this study are available on request from the corresponding author due to privacy and ethical constraints.
