# Correction: Yamamoto et al. Investigation of Oral Health Awareness and Associated Factors Among Japanese University Students: Analyzing Behaviors Influencing Lifelong Oral Health Promotion. *Healthcare* 2025, *13*, 1370

**DOI:** 10.3390/healthcare14101404

**Published:** 2026-05-20

**Authors:** Tsukasa Yamamoto, Manato Seguchi, Yukihiro Mori, Harumi Ejiri, Mamoru Tanaka, Hana Kozai, Yoko Iio, Yuka Aoyama, Morihiro Ito

**Affiliations:** 1 Graduate School of Life and Health Sciences, Chubu University, 1200 Matsumoto-cho, Kasugai-shi 487-8501, Aichi, Japan; 2 Center for Nursing Practicum Support, Chubu University, 1200 Matsumoto-cho, Kasugai 487-8501, Aichi, Japan; 3 Support for Pioneering Research Initiated by the Next Generation (SPRING), Chubu University, 1200 Matsumoto-cho, Kasugai-shi 487-8501, Aichi, Japan; 4 Department of Nursing, College of Life and Health Sciences, Chubu University, 1200 Matsumoto-cho, Kasugai-shi 487-8501, Aichi, Japan; 5 Department of Food and Nutritional Sciences, College of Bioscience and Biotechnology, Chubu University, 1200 Matsumoto-cho, Kasugai-shi 487-8501, Aichi, Japan; 6 Department of Lifelong Sports and Health Sciences, College of Life and Health Sciences, Chubu University, 1200 Matsumoto-cho, Kasugai-shi 487-8501, Aichi, Japan; 7 Department of Clinical Engineering, College of Life and Health Sciences, Chubu University, 1200 Matsumoto-cho, Kasugai-shi 487-8501, Aichi, Japan; 8 Department of Biomedical Sciences, College of Life and Health Sciences, Chubu University, 1200 Matsumoto-cho, Kasugai-shi 487-8501, Aichi, Japan

**Error in References**

In the original publication [1], there was an error in the numbering of the references cited in the main text.

In Section 2.2.2, paragraph 2, “Rama et al. [18] and Ishikawa et al. [19]” was incorrectly stated. The correct wording is “Ishikawa et al. [18] and Rana et al. [19]”, reflecting both corrected author names and reference numbering.

**Error in Tables**

Due to data transcription mistakes during table preparation, there were also several mistakes in the tables as published.

First, in Tables 1 and 2, under “Year of study”, the percentage for “Third year” was incorrectly stated as “20.4%”. The correct value is “20.5%”.

Next, in Table 2, under “Satisfaction with current life”, the percentage for “Not satisfied” in the overall column was incorrectly stated as “23.5%”. The correct value is “26.5%”.

The corrected tables appear below.

**Table 1:** Summary of participant characteristics.

				*n* = 5482
		*n*		%
Mean age ± SD	19.32 ± 1.26			
Sex				
Male		3693		67.4%
Female		1752		32.0%
No answer		37		0.7%
Year of study				
First year		2176		39.7%
Second year		1175		21.4%
Third year		1121		20.5%
Fourth year		1010		18.4%
Living together with family				
Living together		4623		84.3%
Not living together		859		15.7%
Varsity sport participation				
Yes		720		13.1%
No		4762		86.9%
Smoking history				
Yes		382		7.0%
No		5100		93.0%
Satisfaction with current life				
Satisfied		4031		73.5%
Not satisfied		1451		26.5%
Overall health				
Good		4975		90.8%
Poor		507		9.2%

SD, standard deviation.

**Table 2:** Relationship between attributes and oral health.

Variables	All		Oral Health				*p*-Value
			Poor *n* = 1323, 24.1%		Good *n* = 4159, 75.9%		
	*n*	%	*n*	%	*n*	%	
**Attribute**							
Age							
18–19 years	3215	58.6%	769	58.1%	2446	58.8%	n.s.
21 years and older	2267	41.4%	554	41.9%	1713	41.2%	
Sex							
Male	3693	67.4%	901	68.1%	2792	67.1%	n.s.
Female	1752	32.0%	414	31.3%	1338	32.2%	
No answer	37	0.7%	8	0.6%	29	0.7%	
Year of study							
First year	2176	39.7%	495	37.4%	1681	40.4%	n.s.
Second year	1175	21.4%	306	23.1%	869	20.9%	
Third year	1121	20.5%	284	21.5%	837	20.1%	
Fourth year	1010	18.4%	238	18.0%	772	18.6%	
Living together with family							
Living together	4623	84.3%	1127	85.2%	3496	84.1%	n.s.
Not living together	859	15.7%	196	14.8%	663	15.9%	
Varsity sport participation							
Yes	720	13.1%	183	13.8%	537	12.9%	n.s.
No	4762	86.9%	1140	86.2%	3622	87.1%	
Smoking history							
Yes	382	7.0%	98	7.4%	284	6.8%	n.s.
No	5100	93.0%	1225	92.6%	3875	93.2%	
Satisfaction with current life							
Satisfied	4031	73.5%	868	65.6%	3163	76.1%	<0.001
Not satisfied	1451	26.5%	455	34.4%	996	23.9%	
Overall health							
Good	4975	90.8%	1083	81.9%	3892	93.6%	<0.001
Poor	507	9.2%	240	18.1%	267	6.4%	

The *p*-values were calculated using a chi-square test. n.s., no significant difference.

Furthermore, in Table 4, under “Interest in oral health”, the percentages for “Yes/No” in the overall column were incorrectly listed as “(Yes) 81.5%, (No) 18.5%”. The correct values are “(Yes) 72.2%, (No) 27.8%”.

The corrected table appears below.

**Table 4:** Relationship between awareness of oral health and oral health status.

Variables	All		Oral Health				*p*-Value
			Poor *n* = 1323, 24.1%		Good *n* = 4159, 75.9%		
	*n*	%	*n*	%	*n*	%	
**Awareness of Oral Health**							
Interest in oral health							
Yes	3960	72.2%	970	73.3%	2990	71.9%	n.s.
No	1522	27.8%	353	26.7%	1169	28.1%	
Interest in oral hygiene							
Yes	4468	81.5%	1059	80.0%	3409	82.0%	n.s.
No	1014	18.5%	264	20.0%	750	18.0%	
Knowledge of the 8020 campaign							
Yes	3246	59.2%	737	55.7%	2509	60.3%	0.003
No	2236	40.8%	586	44.3%	1650	39.7%	
Knowledge that periodontal disease is a lifestyle disease							
Yes	4272	77.9%	1017	76.9%	3255	78.3%	n.s.
No	1210	22.1%	306	23.1%	904	21.7%	
Knowledge of the impact of smoking on periodontal disease development							
Yes	4302	78.5%	981	74.1%	3321	79.9%	<0.001
No	1180	21.5%	342	25.9%	838	20.1%	
Consciously selected toothbrushing tools							
Yes	1717	31.3%	303	22.9%	1414	34.0%	<0.001
No	3765	68.7%	1020	77.1%	2745	66.0%	
Tooth brushing with attention to caries and periodontal disease							
Yes	3454	63.0%	731	55.3%	2723	65.5%	<0.001
No	2028	37.0%	592	44.7%	1436	34.5%	
Anxiety about the cost of dental visits							
Yes	1995	36.4%	637	48.1%	1358	32.7%	<0.001
No	3487	63.6%	686	51.9%	2801	67.3%	
Anxiety about pain during dental treatment							
Yes	995	18.2%	386	29.2%	609	14.6%	<0.001
No	4487	81.8%	937	70.8%	3550	85.4%	

The *p*-values were calculated using a chi-square test. n.s., no significant difference.

In addition, in Table 5, under “Frequency of tooth brushing (times/day)”, the data and percentages for poor/good oral health status were incorrectly listed. The corrected values are as follows: 0–1 times/day “Poor: 327, 24.7%; Good: 501, 12.0%”, 2 times/day “Poor: 787, 59.5%; Good: 2676, 64.3%”, and 3 times/day or more “Poor: 209, 15.8%; Good: 982, 23.6%”.

Moreover, in Table 5, under “Water intake (mL/day)”, the percentages in the total column were reversed. The correct values are “(Less than 1000 mL) 54.5%” and “(1000 mL or more) 45.5%”.

The corrected table appears below.

**Table 5:** Relationship between oral health behavior and oral health status.

Variables	All		Oral Health				*p*-Value
			Poor *n* = 1323, 24.1%		Good *n* = 4159, 75.9%		
	*n*	%	*n*	%	*n*	%	
**Oral Health Habits**							
Frequency of tooth brushing (times/day)							
0–1 times/day	828	15.1%	327	24.7%	501	12.0%	<0.001
2 times/day	3463	63.2%	787	59.5%	2676	64.3%	
3 times/day or more	1191	21.7%	209	15.8%	982	23.6%	
Use of mouthwash							
Yes	904	16.5%	174	13.2%	730	17.6%	<0.001
No	4578	83.5%	1149	86.8%	3429	82.4%	
Use of interdental brush and dental floss							
Yes	856	15.6%	184	13.9%	672	16.2%	n.s.
No	4626	84.4%	1139	86.1%	3487	83.8%	
Dental visit (within 6 months)							
Yes	2170	39.6%	514	38.9%	1656	39.8%	n.s.
No	3312	60.4%	809	61.1%	2503	60.2%	
Regular dental visits							
Yes	1540	28.1%	331	25.0%	1209	29.1%	0.004
No	3942	71.9%	992	75.0%	2950	70.9%	
Availability of a family dentist							
Yes	3039	55.4%	717	54.2%	2322	55.8%	n.s.
No	2443	44.6%	606	45.8%	1837	44.2%	
Whether the patient has received education on oral hygiene							
Yes	3485	63.6%	886	67.0%	2599	62.5%	0.003
No	1997	36.4%	437	33.0%	1560	37.5%	
**Eating Habits**							
Frequency of breakfast intake (days/week)							
More than 5 days	3603	65.7%	774	58.5%	2829	68.0%	<0.001
Less than 4 days	1879	34.3%	549	41.5%	1330	32.0%	
Water intake (mL/day)							
Less than 1000 mL	2986	54.5%	730	55.2%	2256	54.2%	n.s.
1000 mL or more	2496	45.5%	593	44.8%	1903	45.8%	
Types of beverages most often consumed							
Water	2747	50.1%	645	48.8%	2102	50.5%	n.s.
Green tea	2506	45.7%	596	45.0%	1910	45.9%	
Soft drinks	229	4.2%	82	6.2%	147	3.5%	
Frequent gum chewing							
Yes	799	14.6%	183	13.8%	616	14.8%	n.s.
No	4683	85.4%	1140	86.2%	3543	85.2%	

The *p*-values were calculated using a chi-square test. n.s., no significant difference.

Finally, in Table 6A, under “Living together with family”, the row labels in the overall column were reversed. The correct order is “Not living together” and “Living together”.

The corrected table appears below.

**Table 6:** (**A**) Oral health behavior—comparison by year of study.

Variables	All		Year of Study								*p*-Value
			First Year *n* = 2176, 39.7%		Second Year *n* = 1175, 21.4%		Third Year *n* = 1121, 20.5%		Fourth Year *n* = 1010, 18.4%		
	*n*	%	*n*	%	*n*	%	*n*	%	*n*	%	
(**A**)											
**Attribute**											
Sex											
Male	3693	67.4%	1497	68.8%	832	70.8%	734	65.5%	630	62.4%	<0.001
Female	1752	32.0%	668	30.7%	335	28.5%	379	33.8%	370	36.6%	
No answer	37	0.7%	11	0.5%	8	0.7%	8	0.7%	10	1.0%	
Living together with family											
Not living together	859	15.7%	309	14.2%	193	16.4%	196	17.5%	161	15.9%	n.s.
Living together	4623	84.3%	1867	85.8%	982	83.6%	925	82.5%	849	84.1%	
Varsity sport participation											
Yes	720	13.1%	288	13.2%	133	11.3%	163	14.5%	136	13.5%	n.s.
No	4762	86.9%	1888	86.8%	1042	88.7%	958	85.5%	874	86.5%	
Smoking history											
Yes	382	7.0%	9	0.4%	27	2.3%	155	13.8%	191	18.9%	<0.001
No	5100	93.0%	2167	99.6%	1148	97.7%	966	86.2%	819	81.1%	
Satisfaction with current life											
Satisfied	4031	73.5%	2027	93.2%	992	84.4%	474	42.3%	538	53.3%	<0.001
Not satisfied	1451	26.5%	149	6.8%	183	15.6%	647	57.7%	472	46.7%	
Overall health											
Good	4975	90.8%	2030	93.3%	1053	89.6%	976	87.1%	916	90.7%	<0.001
Poor	507	9.2%	146	6.7%	122	10.4%	145	12.9%	94	9.3%	

**Error in Ethics Approval**

The Ethics Approval number was incorrectly stated as “20220002”. The correct number is “20220001”. The corrected Institutional Review Board Statement is “This study was conducted in accordance with the principles of the Declaration of Helsinki and conducted with the approval of the Ethical Review Committee of Chubu University (Approval No.: 20230056) (Approval date: 4 October 2023) (Approval No.: 20220001) (Approval date: 12 April 2022).”

The authors apologize for any inconvenience caused. The authors state that the scientific conclusions are unaffected. This correction was approved by the Academic Editor. The original publication has also been updated.
